# Evaluating the Quality of National Mortality Statistics from Civil Registration in South Africa, 1997–2007

**DOI:** 10.1371/journal.pone.0064592

**Published:** 2013-05-27

**Authors:** Jané Joubert, Chalapati Rao, Debbie Bradshaw, Theo Vos, Alan D. Lopez

**Affiliations:** 1 School of Population Health, The University of Queensland, Herston, Brisbane, Queensland, Australia; 2 Burden of Disease Research Unit, South African Medical Research Council, Parow Vallei, Western Cape, South Africa; 3 Institute of Health Metrics and Evaluation, University of Washington, Seattle, Washington, United States of America; 4 Melbourne School of Population and Global Health, The University of Melbourne, Carlton, Victoria, Australia; London School of Economics, United Kingdom

## Abstract

**Background:**

Two World Health Organization comparative assessments rated the quality of South Africa’s 1996 mortality data as low. Since then, focussed initiatives were introduced to improve civil registration and vital statistics. Furthermore, South African cause-of-death data are widely used by research and international development agencies as the basis for making estimates of cause-specific mortality in many African countries. It is hence important to assess the quality of more recent South African data.

**Methods:**

We employed nine criteria to evaluate the quality of civil registration mortality data. Four criteria were assessed by analysing 5.38 million deaths that occurred nationally from 1997–2007. For the remaining five criteria, we reviewed relevant legislation, data repositories, and reports to highlight developments which shaped the current status of these criteria.

**Findings:**

National mortality statistics from civil registration were rated satisfactory for coverage and completeness of death registration, temporal consistency, age/sex classification, timeliness, and sub-national availability. Epidemiological consistency could not be assessed conclusively as the model lacks the discriminatory power to enable an assessment for South Africa. Selected studies and the extent of ill-defined/non-specific codes suggest substantial shortcomings with single-cause data. The latter criterion and content validity were rated unsatisfactory.

**Conclusion:**

In a region marred by mortality data absences and deficiencies, this analysis signifies optimism by revealing considerable progress from a dysfunctional mortality data system to one that offers all-cause mortality data that can be adjusted for demographic and health analysis. Additionally, timely and disaggregated single-cause data are available, certified and coded according to international standards. However, without skillfully estimating adjustments for biases, a considerable confidence gap remains for single-cause data to inform local health planning, or to fill gaps in sparse-data countries on the continent. Improving the accuracy of single-cause data will be a critical contribution to the epidemiologic and population health evidence base in Africa.

## Introduction

The importance of reliable, valid, comparable mortality data for measuring and improving population health is widely acknowledged, [1], [2] yet few low and middle income countries have such data, [1], [3] and even fewer have assessed the quality of their mortality data. [3] Periodic evaluations of mortality statistics by countries themselves are particularly useful to identify biases in local data, to shed light on the causes or sources of these biases, and to make locally-relevant recommendations to address these. [3], [4] Among the few countries that have carried out such an exercise in the developing world are India, [5] China, [4] and Brazil [6].

A comprehensive country-specific evaluation of national mortality statistics has not yet been conducted for South Africa. However, based on 1996 data, two WHO assessments [1], [3] rated South Africa in a group of countries with low quality mortality data, using as criteria completeness of death registration (<70%), use of ill-defined codes (>20% of registered deaths), timeliness and/or using an old revision of the International Statistical Classification of Diseases and Related Health Problems (ICD) or alternate list to code causes of death.

Although death registration was enacted in 1867 and the national statistical office established in 1914, partial coverage driven by variations in civil registration practices for different geographic areas and population groups, resulted in limited utility of national vital statistics for most of the twentieth century. [7]–[9] Under the Population Registration Act of 1950, South Africans were classified as ‘Black African’, ‘Coloured’, ‘Indian/Asian’ or ‘White’. This population group classification was associated with disparities in different spheres of life, including civil registration practice, mobility, and residential access, all constraining the coverage of civil registration and completeness of death reporting.

With the emergence of democracy in the early 1990s, governance and public services, including civil registration, underwent major transformation through changing legislation, policy and practice. [10] Three key events played a major role to facilitate improvements in coverage and content of civil registration: (i) the passing of the Births and Deaths Registration Act of 1992, abolishing differential vital registration based on race and rural residence; (ii) the adoption of the Interim Constitution of South Africa in 1993, consolidating the geographically-segmented country into one geo-political unit which enabled the centralisation of the civil registration and vital statistics system (later confirmed in the Final Constitution of 1996); and (iii) a collaboration among strategic role players, i.e. the Department of Health, Department of Home Affairs, Statistics South Africa (StatsSA) and mortality researchers, focussing on enhancing the vital statistics system [8].

A range of initiatives followed these events, towards improving birth and death registration practice and the quality of vital data. These included the introduction of a new death notification form (DNF) that complies with WHO standards; establishment of provincial task teams to assist in implementing the new DNF; distribution of certification and ICD coding manuals for health staff; and guidelines on birth and death registration. [10] In addition, letters about relevant guidelines and new procedures were issued to all registered doctors; and birth registration forms were made available to mothers at the time of delivery, with health workers trained in all provinces to assist mothers to complete and submit these forms. [10] Finally, targeted capacity development was undertaken at StatsSA, to improve compilation and production of annual vital statistics for South Africa [8], [10].

Given these initiatives, it is of considerable interest to ascertain whether or not they have had an impact on the quality of more recent civil registration data. Furthermore, South Africa’s cause-specific mortality patterns have previously been used to model mortality in many sub-Saharan Africa countries [11] and continue to be used by research and international development agencies as the basis for making estimates of cause-specific mortality in many African countries since there is very little information on mortality patterns elsewhere. [12] This adds to the critical importance of understanding the quality of South Africa’s mortality data. We therefore aim to evaluate the quality of national mortality statistics from civil registration for the period 1997–2007, to report areas of data strengths and weaknesses, and to make recommendations for improvements in data quality.

## Methods

### Ethics Statement

The mortality data used in this study were obtained from the official statistics agency of South Africa, StatsSA, collecting and providing data under the provisions of the Statistics Act of 1999. [13] To obtain mortality rates, we used population data from a publically-available electronic data source of the Actuarial Society of South Africa (ASSA). [14] Ethical clearance for research involving human participants was not sought as the datasets are anonymous and contain no identifiable information of any study participant. Ethical concerns regarding participant consent and possible negative consequences to study participants have been taken note of, but are not relevant to the study as the study ‘participants’ are deceased persons. In interactions with collaborators in the host country, however, relevant ethics considerations such as respect for local customs and legal requirements regarding data-use have been upheld.

### Assessment Framework

In the past, completeness of death registration was commonly the only assessment criterion applied to evaluate the quality of national vital statistics. [3] However, as awareness of the usefulness of cause-of-death statistics increased, more assessment criteria were proposed. [15] These criteria have been expanded and used in a framework of which the origin [5], [15] and conceptual underpinnings [4], [6] have been described elsewhere. To evaluate South Africa’s mortality data, we built on earlier country-specific evaluations, [4]–[6] employing the general attributes and criteria as defined in the China study [4]:

- Generalizability, informed by the criteria (1) coverage and (2) completeness of death registration;
- Reliability: (3) epidemiological consistency and (4) temporal consistency;
- Validity: (5) content validity, (6) use of ill-defined and non-specific codes, and (7) use of age- and sex-improbable classifications; and
- Policy relevance: (8) timeliness, and (9) availability of sub-national data.

Each criterion is rated with three broadly-defined evaluation measures: “satisfactory”, “unsatisfactory” or, where the information is unavailable or insufficient, “unknown”. For differentiating between “satisfactory” and “unsatisfactory”, we employ the thresholds suggested in previous studies [4], [6].

### Data Sources

For five criteria (*coverage*, *completeness*, *timeliness*, *sub-national availability* and *content validity*) information was reviewed in relevant legislation, statistical releases, web-based data repositories, research and government reports, and scholarly journals to inform about developments over time which shaped the current status of these criteria in terms of data adequacy.

For the remaining four criteria (*epidemiological consistency*, *temporal consistency*, *age/sex classification*, and *ill-defined/non-specific codes*) the evaluation draws on a dataset produced by StatsSA with 11 years’ mortality data from DNFs for 5.38 million deaths that occurred nationally from 1 January 1997 to 31 December 2007. [16] This dataset comprises of deaths certified according to the following practices. In cases of natural deaths with access to a medical practitioner, the 1992 Act requires the practitioner to complete a DNF (Form BI-1663). The DNF also makes provision for a registered professional nurse to do so. If neither is available, as may happen for example in remote rural areas, a Death Report (From BI-1680) must be completed by an authorized traditional leader (headman/chief), member of the police service, or funeral undertaker to certify the death and describe the circumstances that led to the death. [17], [18] Unnatural deaths are subject to medico-legal investigation in terms of the Inquests Act of 1959. On receipt of the DNF or Death Report by the Department of Home Affairs, the death is registered into the electronic civil registration system. Hereafter, the forms are collected by StatsSA where trained nosologists code all causes to ICD-10 3-digit codes. [19] Underlying causes are derived automatically with the Automated Classification of Medical Entities software (ACME 2000.05) [20].

### Assessing the General Attributes and Criteria

**Generalisability**, or the extent to which mortality statistics are representative of the population under study, was assessed using the criteria *coverage* and *completeness*. *Coverage* refers to the extent of inclusion of different sectors of the population in the civil registration system, such as geographical sectors (e.g. urban/rural, or sample-based areas); administrative sectors (e.g. provinces, states or districts); or population groups based on country-specific categorizations. *Completeness* refers to the extent to which deaths within the covered population are reported into the civil registration system. For *coverage*, we reviewed and summarised the effect of legislation and policies that mandated and/or constrained geographic, administrative and population coverage of death registration over the past 150 years. Due to unrepresentativeness of the total population and the potential of introducing biases into the data, *coverage* of less than the total population is deemed ‘unsatisfactory’. For *completeness*, published estimates of under-registration of deaths were reviewed. Because of the need to measure the patterns and rates of mortality in a population with the minimum biases, completeness of less than 90% of the covered population is rated ‘unsatisfactory’.

**Reliability** relates to the consistency of mortality data with regard to established epidemiological expectations. For this general attribute, we evaluated two criteria: *epidemiological consistency* and *temporal consistency*. *Epidemiological consistency* of the South African data was evaluated using methods similar to those used in previous country evaluations of national vital registration systems, [4], [6] and a variation thereof. Based on the premise that the composition of mortality by cause changes systematically as all-cause mortality decline, [21]–[23] observed broad patterns of causes of death were compared with expected broad-cause values considering the relationship between the overall level of mortality and the relative contribution of causes to the overall level. The country’s gross domestic product (GDP) is used as a covariate in the model. Such evaluation is based on the theory of the epidemiological transition, according to which declines in all-cause mortality are accompanied by shifts in proportionate mortality: in high-mortality populations, communicable, reproductive and nutritional conditions predominate, whereas chronic and degenerative conditions predominate in low-mortality populations. [21] A historical dataset of international vital registration data was analysed by Salomon and Murray [23] to develop regression models that predict cause-specific compositional mortality by broad cause groups, for given inputs of all-cause mortality by age and sex. The three broad-cause groups are (1) a combined group of communicable diseases, maternal, neonatal and nutritional causes, (2) non-communicable disease, and (3) injuries, as defined in the Global Burden of Disease 1990 study [24].

To assess *epidemiological consistency*, the model predictions by age, sex and broad cause were compared with observed proportions for South Africa. A difference of more than two standard deviations (>2 SD) between observed and predicted proportions suggests unsatisfactory epidemiological consistency of the observed data, unless there are plausible epidemiological reasons for such departures. [4] We used national mortality data by age and sex from civil registration for 2007; population estimates for 2007 from the ASSA2008 AIDS and Demographic Model (ASSA2008) of ASSA; [14] and 2007 GDP estimates from StatsSA [25] to derive model-predicted broad-cause proportionate mortality by age and sex. At first, we compared the broad-cause proportions derived from the cause-of-death models with observed proportionate mortality for South Africa. However, as the compositional cause of death models are based on mortality schedules from countries and time periods not affected by HIV/AIDS, we also compared the model-based predictions with observed broad-cause proportions after excluding from the observed data the large numbers of death due to HIV/AIDS for 2007 as estimated in preparation for the second South African National Burden of Disease study [26].

*Temporal consistency* was evaluated by examining whether proportionate mortality from 10 leading causes or cause-groups changed in a predictable manner over time in the period 1997 to 2007. This criterion is informed by the proposition that proportionate mortality from different causes changes in a predictable manner over time as overall mortality changes with socio-economic development. [21], [23] In the absence of substantial natural disasters, pandemics, or revisions to the classification of diseases, a consistent trend in cause-specific mortality should be observed. Where such impacts occurred, as in the case of the substantive HIV/AIDS epidemics in sub-Saharan Africa, observed cause-specific mortality trends would be expected to reflect increased deaths resulting from the epidemic. We investigated the trajectory over time of malignant neoplasms, ill-defined natural causes, external causes, and infectious and parasitic disease which were among the most commonly-reported categories or groups of disease during the 11-year period. We also examined tuberculosis, lower respiratory infections, diarrhoeal disease, ischaemic heart disease (IHD), stroke, and diabetes, counting among the most commonly-reported communicable and non-communicable single causes for 1997–2007 and ranking among the 10 leading single causes in the South African National Burden of Disease Study, 2000 [27].

For the attribute **validity**, we sought to assess the extent to which mortality data show what they purport to show, and to assess the extent of insufficiently- and inappropriately-attributed causes of death. Three criteria were assessed based on information on DNFs. *Content validity* (criterion 5) was assessed by reviewing local studies that examined the accuracy of cause attribution. Like inaccurate cause attribution, the *use of ill-defined or non-specific codes* (criterion 6) is a large impediment to local usefulness and international comparison of cause data. A proportion larger than 10% of total deaths assigned to ill-defined or non-specific codes was considered unsatisfactory. Aggregated data for 1997–2007, nationally and by province of death occurrence, were analysed to identify the extent of Chapter R codes (Symptoms, signs and ill-defined conditions); three non-specific cancer codes (C76, C80, C97); two major ill-defined cardio-vascular disease (CVD) causes (heart failure (I50) and cardiac arrest (I46)); and injuries of undetermined intent (Y10–Y34). Additionally, to compare the extent of R codes by age, R codes in each age group were calculated as a percentage of total deaths in each age group. Finally, the number of deaths coded to R codes was calculated by province for each year to compare the trajectory of R codes to that of the total number of deaths over the eleven years for each province.

Criterion 7, *use of age- and sex-improbable classification*, is guided by the observation that certain conditions occur primarily in specific age ranges, or cause sex-specific mortality. Departures from anticipated age/sex patterns raise concern about the quality of cause data. The aggregate dataset was examined for departures from 10 sex-specific conditions comprising maternal causes of death and genital tract cancers (Text S1). Age patterns were examined for plausibility and consistency in 27 typically age-dependent causes/cause groups: maternal conditions, perinatal conditions, 16 cancers, cardiovascular disease, and suicide (Text S1). In addition, unadjusted annual age-specific death rates were calculated over the 11-year period for three leading cause groups, i.e. cerebrovascular disease, malignant neoplasms, and IHD, to assess plausibility across age from the raw data. Patterns of age- and sex-specific rates were examined from the aggregated unadjusted deaths from cerebrovascular disease by province of death occurrence, and nationally, to assess age-consistency across the provinces.

**Policy relevance** was evaluated by assessing *timeliness* of the release of mortality data (criterion 8) and *availability of sub-national data* (criterion 9). These criteria, respectively, are informed by the proposition that out-of-date mortality data are of little relevance for policy and intervention purposes, and that nationally-aggregated data are insufficient to identify local health differentials and needed interventions by health jurisdiction. *Timeliness* was assessed by examining the time gap between the end of the reference period (year of death) and the time of publication of final tabulations. A lag of two years was considered a reasonable threshold. [4] Criterion 9 was evaluated by assessing the public availability of geographically-disaggregated data in paper and electronic reports, online data repositories, and unit record data, at least at provincial level.

## Results

### Criterion 1: Coverage

Death registration, enacted since 1867, was effectively a partial process for most of the 1900s as coverage was constrained by differential registration practice based on geographical segmentation and population segregation imposed by various acts under ‘homeland’ and apartheid policies. [8], [28] During the 1990s, the ‘homeland’ ideology was abolished under democratic rule, and the country was geographically unified under one government. With the Births and Deaths Registration Act of 1992, death notification became a national, inclusive legal requirement for all people in all geographic areas. Hence, *coverage* is rated satisfactory.

### Criterion 2: Completeness

**Figure 1:**
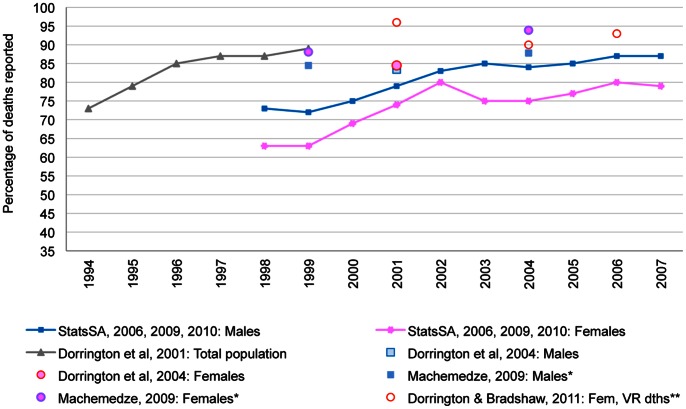
Estimated completeness of death reporting in South Africa: 1994–2007. Notes: *1999 and 2004 are used as midpoints between Census 1996 and Census 2001, and Census 2001 and Community Survey 2007, respectively. **Estimates apply to ages 5–85 years. 2004 was used as midpoint for the period 2001–2007. While the source [33] includes separate GGB and SEG estimates, this graph shows an average of GGB and SEG estimates combined. GGB – Generalized Growth Balance Method; SEG – Synthetic Extinct Generations Method. **Source**: Compiled by the authors from: Stats SA, 2006, [41] 2009, [40] 2010; [17] Dorrington et al., 2001; [31] Dorrington et al., 2004; [32] Machemedze, 2009; [43] Dorrington & Bradshaw, 2011 [33].

Until the 1980s, national completeness of death registration was largely unknown. [29] However, pivotal work has been done by mortality researchers during the 1980s and 1990s, using different methods and data-sources in search of plausible results. [29]–[33] After consolidating fragmented data sources, and taking into account the heterogeneity of the age structures of the four population groups, distortions introduced into the data by the rapidly- but differentially-spreading HIV/AIDS epidemic, and administrative complications of the data, Dorrington et al. [31] estimated national completeness. Applying Bennett and Horiuchi’s Synthetic Extinct Generations Method (SEG) [34] with deaths from civil registration and the Population Register, relative to population estimates from the ASSA600 AIDS model, [35] large improvements were observed in national completeness of adult deaths, increasing from 73% in 1994 to 89% in 1999/2000. [31] (Fig. 1).

A new opportunity to estimate completeness arose in 2001 with the second all-inclusive census under democracy. [36] Using population counts from the 1996 and 2001 censuses, and inter-censal deaths from civil registration, Dorrington and colleagues [32] estimated completeness with Hill’s Generalized Growth Balance (GGB) [37] method with an adaptation to account for migration. [32] National completeness levels were estimated at 83.4% for males and 84.5% for females. [32] (Fig. 1) In 2007, another nationally-representative data source, the Community Survey, [38] offered further opportunity to assess completeness. Investigating maternal mortality in South Africa, Dorrington and Bradshaw [33] used population counts form the Community Survey and the 2001 census, and deaths from vital registration, and estimated national female completeness for 2001–2007 at 91% with the GGB method, and 89%, using the SEG method (Fig. 1). Furthermore, using the Preston and Hill method [39] with inputs from StatsSA mid-year population estimates and vital registration deaths, StatsSA has estimated completeness to have improved to levels of 87% for males and 79% for females in 2007 [40], [41].

Large improvements have been estimated for registration of childhood deaths. For the period 1996–2006, Darikwa and Dorrington [42] estimated infant death registration to have improved from 43% to 89%; registration for children 1–4 years, from 43% to 57%; and for children under 5 years, from 44% to 78% (childhood completeness not presented in Fig. 1). These estimates were derived using a multi-stage method using registered death data from civil registration, data from the 2007 Community Survey (children ever born/children surviving data, data on the survival of the last child born to women aged 12–49 years; and child deaths over the past 12 months as reported by households); 2001 Census (reported household deaths); and previous research which used mortality data based on the 1998 SADHS and 1996 Census [42].

Figure 1 shows a trend of ongoing improvements over time, starting to satisfy the 90% threshold by the early 2000s, while more recent estimates by Dorrington and Bradshaw [33] and Machemedze [43] exceed 90%. For the Global Burden of Disease Study 2010, Wang and colleagues estimated completeness in South Africa at 95% since 2000, [12] and a satisfactory rating is given for *completeness*.

### Criterion 3: Epidemiological Consistency

**Figure 2:**
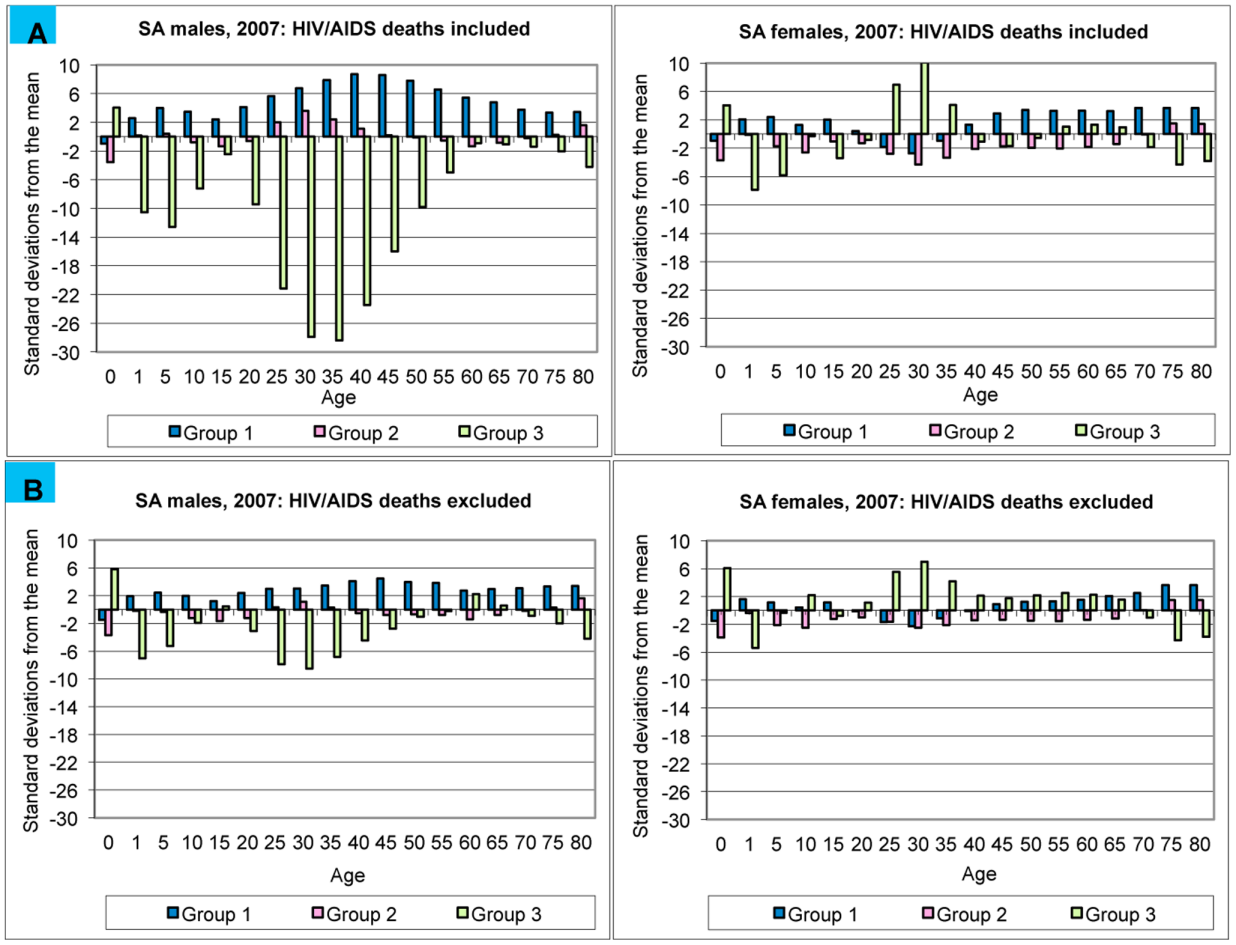
A. Number of standard deviations by age and sex for South Africa, 2007: HIV/AIDS deaths included. The number of standard deviation by which observed broad-cause mortality proportions differ from mean predicted proportions when the estimated number of HIV/AIDS deaths are included in the analysis. **B.** Number of standard deviations by age and sex for South Africa, 2007: HIV/AIDS deaths excluded. The number of standard deviation by which observed broad-cause mortality proportions differ from mean predicted proportions when the estimated number of HIV/AIDS deaths are not included in the analysis. **Source**: Mortality data for 2007 from StatsSA vital registration data; [16] HIV/AIDS estimates from Bradshaw et al.; [26] population data from ASSA2008; [14] GDP data from StatsSA [25].

Large differences were found between the observed and predicted proportional broad-cause mortality patterns for 2007 (Fig. 2A) before taking HIV/AIDS deaths out of the schedule. Considerably better congruence was found between predicted and observed proportions after removing HIV/AIDS deaths (Fig. 2B). However, close to half of male and 41% of female values still deviated by more than two standard deviations from mean predicted proportions in Fig. 2B. These findings have two interpretations. Firstly, the differences in Figures 2A and 2B indicate that HIV/AIDS mortality is not adequately predicted by the cause-of-death models used in this analysis. Secondly, the persistence of deviations after exclusion of HIV/AIDS deaths, particularly for Group 1 conditions in males, could either be a real phenomenon of higher proportionate mortality from infectious diseases, or an artefact of data quality in terms of cause-of-death assignment. On the basis of differences between the South African epidemiological profile and that represented in the model predictions, as well as the observed residual differences on exclusion of HIV/AIDS deaths, this criterion could not be assessed conclusively as the model does not have the discriminatory power to enable an assessment for South Africa.

### Criterion 4: Temporal Consistency

**Figure 3:**
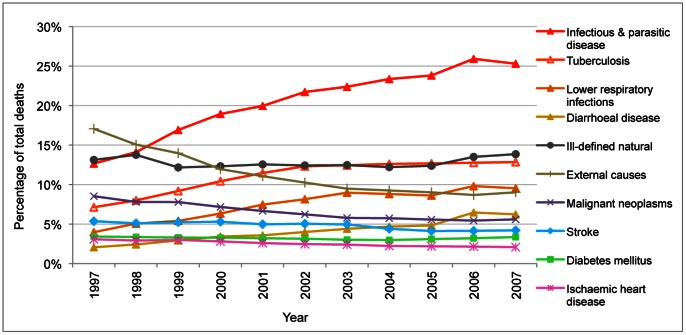
Proportion of total deaths due to leading categories and causes of death, 1997–2007. Source: Vital registration data from StatsSA [16].

Temporally consistent trends are shown over the 11-year period with no large annual fluctuations in cause-specific mortality proportions for IHD, diabetes, stroke, malignant neoplasms and ill-defined natural causes (Fig. 3). Rising proportions of selected communicable diseases are consistent over time and in line with expected epidemiological patterns associated with the HIV/AIDS epidemic and the effect of misclassified HIV/AIDS deaths. [44], [45] Given trends that are consistent and concurring with local epidemiological experiences, the data is rated satisfactory for this criterion.

### Criterion 5: Content Validity

The extent to which certification and coding of causes of death are accurate is not routinely assessed, neither has it been studied in a nationally-representative sample of deaths. However, a few studies have shed light on the accuracy of cause attribution. Most of these studies have focussed on the misclassification of HIV/AIDS deaths, and most were limited in size and geographical coverage. Westwood studied death certificates and medical records at a teaching hospital in Cape Town among 500 paediatric deaths, and found that the under-certification of HIV-related deaths was over 11%, and that a further 30% of HIV-related deaths were classified using non-specific terms that could result in inaccurate classification. [46] Grandin *et al*. reported that, although HIV/AIDS accounted for the leading cause of death, causing 32% of all deaths in their study for 1999 to 2003 in a pediatric teaching hospital in Cape Town, the findings are likely under-estimating the role of HIV in causing death in this setting. [47] Comparing DNFs with medical records for 683 deaths in Cape Town, Yudkin *et al*. estimated that 36% of deaths attributable to HIV were classified to other conditions. A further 37% were indicated by euphemisms such as immune suppression or retroviral disease. [48] In a retrospective review of 242 DNFs of deaths occurring in an academic hospital in Cape Town during 2004, Nojilana *et al*. [49] found that the under-reporting of HIV/AIDS was 53%. Analysing vital registration data for 2000–2001, Groenewald *et al*. found 61% of HIV/AIDS deaths classified to other conditions. [44] Birnbaum *et al*. confirmed substantial misclassification of HIV/AIDS in registered deaths for 1996–2006, suggesting that over 90% of HIV/AIDS deaths were misattributed to other causes. [45] In a recent validation study of 703 deaths in Cape Town, substantial misclassification was found not only for HIV/AIDS, but also for IHD, hypertensive disease, and diabetes. [50] *Content validity* is rated unsatisfactory based on these findings.

### Criterion 6: Use of Ill-defined and Non-specific Causes

Nationally, 12.8% of registered deaths were assigned an R code during 1997–2007, ranging from 12.2% in 1999 to 13.9% in 2007 (Table 1). Typically, about 11% of adult (aged 15–64) deaths had R codes assigned, increasing steadily over the older age groups to double this at ages 80+ (Fig. S1A). Close to two-thirds of R codes were found in persons aged 0–64 years and 36% in ages 65+. Cause-specific proportions from ill-defined and non-specific conditions show substantial differences among the provinces (Table 1). While the number of registered deaths increased dramatically in all nine provinces over time, even doubled in six, the trajectory of Chapter R codes are very different, with much less dramatic increases in absolute numbers (Figures S1B+C). However, with over 10% of causes assigned an R code, and a quarter assigned R- and non-specific codes collectively, this criterion is rated unsatisfactory.

**Table 1:** Percentage of total deaths assigned selected ill-defined and non-specific codes by province of death occurrence, South Africa, 1997–2007.

	Western Cape	EasternCape	Northern Cape	FreeState	KwaZulu-Natal	North West	Gauteng	Mpuma-langa	Limpopo	South Africa
Ch. R codes	6.0	17.5	7.5	9.6	15.0	9.9	12.4	8.5	17.9	12.8
Non-spec. cancer	1.5	0.6	0.7	0.5	0.5	0.4	0.8	0.4	0.4	0.6
Ill-def. CVD	3.0	3.0	4.0	4.5	3.5	5.1	3.8	3.4	3.8	3.7
Ill-def. injury	12.3	6.6	6.3	5.4	6.9	5.7	10.4	7.0	4.4	7.6
All four categories	22.8	27.7	18.5	20.0	25.9	21.1	27.4	19.3	26.5	24.7

Notes: Chapter R codes: R00–R99 (Symptoms, signs and ill-defined conditions); Non-specific cancer codes: C76, C80, C97; Ill-defined cardiovascular disease (CVD) causes: heart failure (I50) and cardiac arrest (I46)); Ill-defined injury: injuries of undetermined-intent, Y10–Y34.

Ch. – Chapter; Non-spec.– Non-specific; Ill-def. – Ill-defined; CVD – Cardiovascular disease.

### Criterion 7: Use of Age- and Sex-improbable Classifications

Departures from uniquely sex-specific causes were found in less than one in a million deaths, and from age-dependent causes, in less than one in two thousand deaths (Text S1). Annual age-specific death rates from the raw data for two leading single causes (stroke and IHD) and one leading cause category (malignant neoplasms) reflect the typical increase with increasing age consistently over the 11-year period. Small inconsistencies appear at ages 80 and over for 1997–1999, possibly reflecting age misreporting, problems with cause attribution in older patients with multiple cause presentation, or lower certification and coding accuracy during earlier years of the reference period (Fig. S2A). Age and sex patterns of unadjusted stroke deaths by province appear plausible over age, though starting at relatively young ages (30 years). These patterns, however, are consistent over age for both sexes and across the provinces (Fig. S2B). Criterion 7 is therefore rated satisfactory.

### Criterion 8: Timeliness

Prior to 1994, the extraction and processing of mortality data from DNFs were tedious. After recording a death on the population register, images of the DNFs were put on microfilm, microfilmed images were manually verified against the original DNFs, DNFs were manually indexed and then sent to StatsSA for processing. The verification process, in particular, could take very long. [8] With limited staff training, the classification and coding of causes of death also contributed to delays, [51] and substantial hold-ups followed in the production of mortality statistics during the 1990s. For example, up to November 2001, the latest cause-of-death data available were from 1996, and in December 2001, cause data for 1997–2000 were released. Post-democracy, however, delays have been addressed by concerted multi-stakeholder efforts with a shared common vision to enhance vital statistics; a driving process encompassing motivation and learning from expert opinion; and international donor assistance aimed at staff training in several aspects of vital statistics compilation. [8], [17] Over the past decade, further improvements followed with advances in electronic data compilation, advanced training of nosologists by Australian and United States experts, and automation of cause attribution. [8] Shorter waiting times and improved regularity followed, such that, since 2007, annual reporting was done consistently on deaths that occurred in the calendar year two years prior to publication. [52] Hence, *timeliness* is rated satisfactory.

### Criterion 9: Sub-national Availability

South Africa has nine provinces, 46 district municipalities and 231 municipalities. While statistical releases, published in 1999 [53] and 2001, [51] contained limited mortality data by province as the lowest level of disaggregation, recent reports include numerous tabulations by province and district municipality. At provincial level, web-based tables can be created with a number of DNF variables, including underlying cause. The DNF provides information on the place of death occurrence, place of registration of the death, and place of usual residence of the deceased, holding potential for a wide range of mortality and epidemiological analysis at sub-national level. Anonymous unit-record cause data by province are available electronically upon request, subject to data-use agreements. This criterion is thus rated satisfactory.

## Discussion

Civil registration data were rated satisfactory for six of nine criteria. These six indicate satisfactory performance in terms of generalisability and policy relevance, and partial satisfaction with reliability and validity of the data. The two criteria rated unsatisfactory, *content validity* and *use of ill-defined and non-specific codes*, signify considerable shortcomings with cause-of-death data. Information on content validity, particularly for infectious disease mortality, would clarify the performance rating for the remaining criterion, i.e. *epidemiological consistency*. Overall, the findings from this evaluation indicate that while there have been substantial improvements in the performance and quality of selected aspects of civil registration mortality data, attention needs to be paid to cause attribution before the data are deemed optimal for population health assessment and epidemiological research.

Substantial improvement has been observed in the *completeness* of death registration over a short period of less than two decades. There remains a need, though, for focussed attention to improve registration of deaths in children aged 1–4 years. While registration of child deaths is usually lower than that of adult deaths, [54] this can be changed, as demonstrated through the doubling of completeness of infant death registration in the past decade. [42] Further improvements in registering child deaths would be a significant step towards more accurate estimates of under-5 mortality from vital registration, with nominal adjustment. The availability of reliable local measures of under-five mortality is of critical importance for targeted public health interventions to help meet the United Nations Millennium Development Goals 4 & 5 for South Africa.

Despite the overall trend towards higher levels of completeness of adult death registration, the actual estimates continue to reflect differences. The limitations of indirect demographic methods used for estimating completeness are known, [55] largely on account of incompatibility of assumptions underlying the individual methods. For instance, death distribution methods are based on the assumption that the study population is closed to migration. Although adjustments can be made to allow for migration, there is much uncertainty regarding the extent of international migration into South Africa. Additionally, death distribution methods also depend on minimal age misreporting and that completeness is constant by age. While research has found misreporting of older-age mortality data [43] and differential completeness of death registration by age, [33], [42] limited expertise and information exist to adjust for the same. The Preston and Hill method [39] applied by StatsSA is also affected by violation of the assumption of a closed and demographically stable population, as well as potential limitations in the use of population growth rates in the method, not its age distribution. [40], [41] A further problem with this attempt to estimate the completeness on an annual basis is that the calculation is done relative to population estimates, effectively comparing the deaths against assumed modelled estimates of mortality incorporated in the population estimation. Until reliable in/out migration data are available, the misreporting of age eliminated, and completeness of death reporting constant by age, data inputs for indirect completeness estimation will remain deficient.

One solution to this problem lies in the derivation of direct estimates of completeness, based on dual-record or capture-recapture methods. This will require rigorous mortality data collection in censuses, surveys, and demographic surveillance sites, and appropriate data-linkage with civil registration data. Other likely additional data sources for such analysis include mortality audits (such as the Confidential Enquiry into Maternal Deaths or Child Healthcare Problem Identification Programme) and national disease registers (such as the Electronic Tuberculosis Register).

Our findings in terms of differences between observed and predicted broad-cause patterns were expected when assessing *epidemiological consistency*, since the model is largely based on data from countries which do not reflect high HIV/AIDS mortality. [23] When we attempted to compensate for this model characteristic by excluding the estimated HIV/AIDS deaths, better results for epidemiological consistency were produced, however, considerable incongruence remained, particularly in terms of higher than predicted mortality fractions for infectious diseases in males. It is possible that mortality from infectious diseases other than HIV/AIDS occur at higher frequency in South Africa as compared to the historical data, at similar levels of all-cause mortality. This can be confirmed from studies on content validity of death registration data, for deaths coded to infectious diseases. Confirmation of coding in a sample of male deaths from infectious diseases could serve as a plausible epidemiological explanation for the differences in proportionate mortality, and could permit a satisfactory rating on this criterion. However, another explanation could be that the HIV/AIDS deaths were underestimated in the preliminary results of the South African National Burden of Disease study in which misclassified HIV/AIDS diagnoses have been estimated based on the excess mortality observed in the distinct age categories, correlated with the increase in HIV prevalence.

The higher than expected proportionate mortality from injuries for women in South Africa are consistent with reports showing that South African women are subject to high levels of intimate partner violence, femicide and rape-homicide. [56]–[59] More generally, South Africa is known to rank among countries with the highest injury mortality in the world, [60] and these high levels of injury mortality among women were not apparent in the historical data represented by the model, which may explain the differences between observed and model-predicted proportions. It should be borne in mind that these are differences in proportions, and do not reflect a considerably larger number of events. However, these findings give sufficient indication for further exploration, triangulation, and validation of the data to confirm the occurrence of such deaths. The findings also call for sufficient detailing on the DNF to effectively influence the necessary public health action.

In South Africa the epidemiological transition has been radically interrupted, suspended, and likely reversed by the HIV/AIDS epidemic after the transition was steadily on course with all-cause child and adult mortality declining, [14], [54], [61] and NCDs progressively taking a larger share [26], [27], [62] during the 1970s and 1980’s until the 1990s. In addition, injuries have been a substantial factor for a long while in the country’s epidemiological profile, rating among the highest globally. [60] This situation points to fundamental differences between the epidemiological profiles on which the model was based and that of South Africa, which is characterised by the quadruple burden from HIV/AIDS; other infectious/childhood & maternal conditions; NCDs; and injuries. [27] It is therefore likely that the observed departures from predicted patterns are real and that they do not necessarily reflect unsatisfactory quality broad-cause data, but rather point to epidemiological heterogeneity. [63], [64] More information on content validity is required to decide whether the differences between the model predictions and observed data are real or an artefact of data quality, and hence we chose to rate the epidemiological consistency of South African data for 2007 as ‘unknown’.

Our study reveals that there is considerable uncertainty in unit record cause-of-death data which limits the usability for local health planning and resource prioritisation, or to estimate mortality in other countries on the continent with sparse or no cause-of-death data. Problems with accuracy of the certification and coding of causes of death are widespread, even in countries with good-quality cause-of-death data. [65]–[67] In South Africa, researchers identified that low quality of medical certification by doctors, certification by non-medical practitioners, and problems in obtaining causes for injury deaths are key challenges to improving data quality. [68] These are valid immediate challenges, but we also recommend that underlying concerns be addressed, including adequate, continued death certification training of medical students and in-service medical certifiers, along with an emphasis on the usefulness of keeping good-quality medical records. Determined efforts are needed to expand civil registration and health services to remote rural areas with adequate staffing. Changes to the DNF are recommended to include fields to adequately record details for external causes (e.g. drowning, or motor vehicle accident) and apparent manner (e.g. accident, homicide, or suicide) of injury deaths, and the venue (e.g. railway track, private house/yard, construction area) and district of the injury that led to death.

Additionally we recommend examining the feasibility of routine validation of a sample of DNFs with doctors’ notes/hospital records/day hospital or clinic cards at major public health facilities such as tertiary hospitals to evaluate data quality from urban areas, as well as linking DSS and vital registration data via capture-recapture methods to assess completeness of death reporting and reliability of reported causes of death in largely rural areas. We also recommend a large-scale validation of cause-of-death data using DNFs and good-quality hospital records to assess both the accuracy of cause attribution and the effect of health interventions. A national validation study may be daunting given severe shortages of human and financial resources, [69] potential challenges in drawing a representative sample, and limitations identified [49], [50] in comparing DNFs and medical records. It may be more achievable to start at health-district level: strengthening skills, knowledge and capacity in one health district to be transferred to the next, and building on pioneering work recently undertaken to develop cause-of-death profiles for the 52 health districts in the country [70].

Some criteria in the evaluation framework have the potential to assist in evaluating others. For instance, high scores of content validity would help identify whether differences between observed and modelled cause-specific mortality proportions have true epidemiological explanations, or result from poor data quality, thereby assisting in rating epidemiological consistency. Content validity scores would also help clarify possible departures in temporal consistency and age or sex patterns by cause. For example, mortality from stroke is observed to rise from ages as early as age 30. If these deaths are proved to be accurately attributed to cause, such early stroke mortality can be proved, and serve as information for clinical and public health interventions to reduce this burden.

### Study Limitations

The study has limitations. The intended evaluation regarding *epidemiological consistency* could not be assessed conclusively as the model does not have the discriminatory power to enable an assessment under this criterion for South Africa, and the rating for this criterion remains unknown. The sub-national data made available for our analysis are classified by province of death occurrence. While such data enables an understanding of data quality at the point or area of death, it also limits an understanding of mortality patterns at the place of usual residence of the deceased in those cases where the place of death occurrence differs from that of usual residence. With no national validation studies available, we based our unsatisfactory rating for *content validity* on results from mainly small-scale studies, and studies with a focus on HIV/AIDS. It may therefore be argued that this criterion cannot be evaluated appropriately, and should be rated ‘unknown’ rather than ‘unsatisfactory’.

### Conclusion

Improvements over time, and six satisfactory ratings out nine quality assessment criteria, are a tribute to the focused efforts and investments by strategic players in the death registration system over the past two decades. Performance of criteria assessed in earlier evaluations [1], [3] have improved such that it would shift South Africa’s mortality statistics from “low” to “medium-high quality”, using the evaluation categories of Mahapatra *et al*. [3] However, the criteria rated ‘unsatisfactory’ have substantial potential to influence the reliability and validity of mortality data, and point to cause-of-death attribution, in particular, as a vulnerable component in producing good-quality local mortality data.

In previous studies, [1], [3], [61] the low availability of mortality data, weak quality of all-cause mortality data, and the absence of single-cause data from sub-Saharan African countries often stood out. This analysis symbolizes optimism for a region marred by data absences and deficiencies by showing considerable progress from a system paralyzed by disintegration, disparity and delay to one with potential to offer integrated, inclusive and timely sub-national all-cause mortality statistics that could be adjusted and used for demographic and health analysis. Additionally, cause-of-death data, certified and coded according to international standards in a recent ICD revision, are available by regional and socio-demographic disaggregation. However, a considerable confidence gap remains for single-cause data to be used for health planning without skilfully estimating adjustments for biases. This assessment, and other research, [26], [71]–[74] have identified priority actions that the South African civil registration, health, and education and training authorities need to take to significantly improve confidence in its mortality statistics, in particular, to improve the accuracy of national single-cause data. Improving the accuracy of single-cause data will be a critical contribution to the epidemiologic and population health evidence base in Africa.

## Supporting Information

Figure S1 **A.** Chapter R codes (R00–R99) in each age group as a percentage of total deaths in that age group, South Africa, 1997–2007. Source: StatsSA vital registration data. [16] **B.** Trends in the number of registered deaths by province of death, South Africa, 1997–2007. Source: StatsSA vital registration data. [16] **C.** Trends in the number of deaths assigned an R code (R00–R99) by province of death, South Africa, 1997–2007. Source: StatsSA vital registration data [16].
(XLSX)

Figure S2 **A.** Unadjusted age-specific death rates by year of death for three major cause groups, South Africa, 1997–2007. Source: Mortality data from StatsSA vital registration; [16] population data from ASSA2008. [14] **B.** Unadjusted age- and sex-specific rates for cerebrovascular deaths, by province and nationally, 1997–2007: Log scale. Source: Mortality data from StatsSA vital registration; [16] population data from ASSA2008 [14].
(XLSX)

Text S1 Sex-specific and age-dependent causes/cause groups.
(DOCX)
