# Supplementary material for: Evaluating the Quality of National Mortality Statistics from Civil Registration in South Africa, 1997–2007
Source: PLoS One. 2013 May 27;8(5):e64592. doi: 10.1371/journal.pone.0064592 (PMC3664567; doi:10.1371/journal.pone.0064592)
Supplement: Text S1 — Sex-specific and age-dependent causes/cause groups. (DOCX) [file pone.0064592.s003.docx]

**Text S1** Sex-specific and age-dependent causes/cause groups.

The aggregate dataset was examined for departures from 10 sex-specific causes: maternal haemorrhage, maternal sepsis, hypertensive disorders of pregnancy, obstructed labour, abortion, other maternal conditions, cervix uteri cancer, corpus uteri cancer, ovary cancer, and prostate cancer.

Age patterns were examined for plausibility and consistency in 27 typically age-dependent causes/cause groups: maternal conditions; low birth weight; birth asphyxia and birth trauma; other peri-natal conditions; 16 cancers (mouth and oro-pharynx; oesophagus; stomach; colon and rectum; liver; pancreas; trachea, bronchus and lung; melanoma and other skin; breast; cervix uteri; corpus uteri; ovary; prostate; bladder; lymphomas and multiple myeloma; leukaemia); rheumatic heart disease; hypertensive disease; ischaemic heart disease; stroke; inflammatory heart disease; benign prostatic hypertrophy; and self-inflicted injuries.
